# Aberrant palmitoylation caused by a ZDHHC21 mutation contributes to pathophysiology of Alzheimer's disease

**DOI:** 10.1002/alz70855_100962

**Published:** 2025-12-23

**Authors:** Wenwen Li

**Affiliations:** ^1^ Innovation Center for Neurological Disorders and Department of Neurology, Xuanwu Hospital, Capital Medical University, Beijing, Beijing, China

## Abstract

**Background:**

The identification of pathogenic mutations in Alzheimer's disease (AD) causal genes led to a better understanding of the pathobiology of AD. Familial Alzheimer's disease (FAD) is known to be associated with mutations in the APP, PSEN1, and PSEN2 genes involved in Aβ production; however, these genetic defects occur in only about 10–20% of FAD cases, and more genes and new mechanism causing FAD remain largely obscure.

**Method:**

We performed exome sequencing on family members with a FAD pedigree and identified gene variant ZDHHC21 p.T209S. A ZDHHC21T209S/T209S knock‐in mouse model was then generated using CRISPR/Cas9. The Morris water navigation task was then used to examine spatial learning and memory. The involvement of aberrant palmitoylation of FYN tyrosine kinase and APP in AD pathology was evaluated using biochemical methods and immunostaining. Aβ and tau pathophysiology was evaluated using ELISA, biochemical methods, and immunostaining. Field recordings of synaptic long‐term potentiation were obtained to examine synaptic plasticity. The density of synapses and dendritic branches was quantified using electron microscopy and Golgi staining.

**Result:**

We identified a variant (c.999A > T, p.T209S) of ZDHHC21 gene in a Han Chinese family. The proband presented marked cognitive impairment at 55 years of age (Mini‐Mental State Examination score = 5, Clinical Dementia Rating = 3). Considerable Aβ retention was observed in the bilateral frontal, parietal, and lateral temporal cortices. The novel heterozygous missense mutation (p.T209S) was detected in all family members with AD and was not present in those unaffected, indicating cosegregation. ZDHHC21T209S/T209S mice exhibited cognitive impairment and synaptic dysfunction, suggesting the strong pathogenicity of the mutation. The ZDHHC21 p.T209S mutation significantly enhanced FYN palmitoylation, causing overactivation of NMDAR2B, inducing increased neuronal sensitivity to excitotoxicity leading to further synaptic dysfunction and neuronal loss. The palmitoylation of APP was also increased in ZDHHC21T209S/T209S mice, possibly contributing to Aβ production. Palmitoyltransferase inhibitors reversed synaptic function impairment.

**Conclusion:**

ZDHHC21 p.T209S is a novel, candidate causal gene mutation in a Chinese FAD pedigree. Our discoveries strongly suggest that aberrant protein palmitoylation mediated by ZDHHC21 mutations is a new pathogenic mechanism of AD, warranting further investigations for the development of therapeutic interventions.

